# On-Body and Off-Body Communications: A Comparative Study Between Hardware and Simulations

**DOI:** 10.3390/s26082561

**Published:** 2026-04-21

**Authors:** Drishti Oza, Alberto Gallegos Ramonet, Masami Yoshida, Taku Noguchi

**Affiliations:** 1 Graduate School of Information Science and Engineering, Ritsumeikan University, Osaka 567-0871, Japan; 2 Graduate School of Technology, Industrial and Social Sciences of Science and Technology, Tokushima University, Tokushima 770-0855, Japan; 3 College of Information Science and Engineering, Ritsumeikan University, Osaka 567-0871, Japan; ydmasami@fc.ritsumei.ac.jp (M.Y.);

**Keywords:** wireless body area networks, WBAN, Narrowband, medical bands, on-body, hardware implementation, simulation, ns-3, Silicon Labs

## Abstract

The IEEE 802.15.6 standard defines wireless body area networks (WBANs) for communication in, on, and around the human body. However, commercially available hardware platforms that support direct experimental validation of IEEE 802.15.6-oriented WBAN studies remain limited. As a result, much WBAN research still relies on simulations or custom-built transceivers, leaving the practical validity of simulation results uncertain. In this study, we evaluated a configurable radio platform for GMSK-based narrowband WBAN PHY validation in the 420–450 MHz band by comparing theoretical calculations, ns-3 simulation results, and hardware measurements. Evaluations covered both on-body and off-body scenarios at transmit powers from −15 to −25 dBm. Our key findings are as follows: (1) lower transmit power consistently decreases the communication range in both simulated and hardware environments; (2) degradation trends in packet success rate are similar for both environments, supporting simulation credibility; and (3) in the off-body scenario, ns-3 simulations overestimate the communication range by approximately 10 m compared to hardware under identical conditions. The publicly available simulation framework facilitates reproducible WBAN research. Our results confirm that our ns-3 implementation can be used effectively to approximate key GMSK-based WBAN PHY behaviors in realistic conditions while identifying specific differences in range estimates.

## 1. Introduction

Wireless body area networks (WBANs) enable effective communication in, on, and around the human body within a network. The IEEE 802.15.6 standard [1] for WBANs aims to optimize communication both inside and on the human body while minimizing any potential health risks. For instance, it considers methods to reduce the body’s absorption rate to minimize the impact of signals on human health.

WBANs are primarily used in healthcare and medical monitoring [2]. WBANs enable real-time tracking and monitoring of vital signals from wearable and implantable sensors for heart rate, body temperature, glucose levels, and electrocardiogram (ECG) signals. WBAN also supports early diagnosis, remote patient monitoring, and postoperative care [3].

In addition to healthcare applications, WBANs are gaining momentum in sports and fitness for performance analysis and injury prevention [4]. They are also used in consumer wearables [5], rehabilitation systems [6], and supportive systems for the elderly and special needs. WBAN systems are increasingly integrated into everyday life. Many devices we commonly use, such as smartwatches, health rings, and smart glasses, fall under the category of WBANs.

The IEEE 802.15.6 standard provides detailed specifications for WBANs, but implementing WBANs on a large scale remains challenging. This is primarily because of the lack of readily available hardware platforms that comply with the standard. Currently, no commercially available transceivers fully support all the physical layers and medium access control (MAC) modes defined by the IEEE 802.15.6 standard.

Hence, many studies depend on simulation tools such as ns-3 [7], Castalia [8], and MATLAB [9] to evaluate the performance of WBANs. Some of these studies even take into consideration reproducibility and usage in real-life scenarios. However, the reliability of WBAN simulation results is highly dependent on validation against practical hardware measurements. Although prior studies have investigated WBAN hardware implementations, simulation models, or non-WBAN radio platforms, direct comparisons between IEEE 802.15.6-oriented WBAN simulations and configurable hardware measurements under matched PHY conditions remain limited. This gap motivates the present study.

Experimental studies have predominantly utilized commercially available low-power radios designed for other well-known IEEE standards [10]. Although these radios partially align with the WBAN parameters, such as frequency bands and data rates, they do not match the WBAN modulation scheme, which can lead to confusion in the research community. There is therefore uncertainty as to whether the findings derived from using different standards as a basis for WBAN systems can be regarded as reliable for WBAN applications.

The challenge becomes even more pronounced in the context of in-body or implant communication. Practical experiments for an in-body WBAN system encounter significant ethical, medical, and safety constraints. The implementation of experimental devices inside the human body solely for communication testing is generally not permissible. Even in controlled clinical environments, these procedures require extensive regulatory approval and medical supervision. Consequently, a direct experimental evaluation of in-body WBAN communication on human subjects is not feasible in most research settings.

Owing to the limited availability of devices for testing purposes, a practical approach for testing on-body WBANs in hardware is to identify a flexible radio that supports WBAN modulation and frequency bands, along with the ability to customize data rates, channel spacing, bandwidth, and other physical layer (PHY) parameters specified by the IEEE 802.15.6 standard [1].

The main contributions of this study are as follows:

1. Configuration and evaluation of a commercial radio platform that supports key IEEE 802.15.6 narrowband PHY parameters for GMSK-based WBAN experiments.
2. A direct experimental comparison between hardware measurements and an ns-3 WBAN PHY model simulation under realistic on-body and near-body scenarios.
3. Practical validation and tuning of the ns-3 WBAN simulation model through consistency analysis against hardware measurements.

The remainder of this paper is organized as follows. Section 2 provides the background and motivations for this study. Section 3 explains the WBAN standard and details the hardware configuration. Section 4 describes the experimental setup and evaluates the hardware results in comparison to the simulation. Finally, Section 6 concludes the paper.

## 2. Background and Motivation

Numerous studies have implemented WBANs on hardware. Some studies have even developed their own IEEE 802.15.6 transceiver for experimentation purposes. One such study was conducted by Kasun et al. [11], who detailed a hardware implementation of an impulse radio ultrawideband (IR-UWB) coordinator node designed for WBAN applications. The work focused on creating a low-power IR-UWB transceiver. They measured the bit error rate (BER) to analyze performance in an on-body to off-body communication scenario at a propagation distance of 1 m. Reproducing or using the results of this study as a basis for future work is complicated, as it requires building the transceiver developed by Kasun et al.

Another study that could be difficult to reproduce was by Xiaonan et al. [12], who implemented IEEE 802.15.6 on hardware, where a WBAN node consisted of a sensor board, a digital signal processor, a field programmable gate array (FPGA), a radio frequency board, and an antenna. They tested the packet and frame error rates at a frequency of 2.4 GHz across different wireless transmission distances and payload lengths. While they considered a WBAN standard complaint data transmission rate of 121.4 kbps for 2.4 GHz, they did not specify other PHY parameters, such as modulation type, preamble length, and header size.

Some studies have used datasets to simulate real-life scenarios. Isak et al. [13] conducted a study that reviewed the energy efficiency and packet loss in transmission power control algorithms for WBANs. This study compared the path loss in real and simulated scenarios, where the real scenario data were derived from an empirical dataset. The experiments in this study were limited to the scenarios in the datasets. Although the study compared real and simulated scenarios, no actual physical hardware was implemented.

Alternatively, some studies have utilized commercially available hardware designed for IEEE standards other than IEEE 802.15.6. One such study was performed by Woosik et al. [10]. The study examined the characteristics of two radio modules, CC2420 and CC1000 (Texas Instruments, Dallas, TX, USA), within wireless body sensor network systems. Their study measured the average received signal strength indication (RSSI), transmission power levels, and the impact of body movement on radio transmission. The hardware considered did not adhere to the WBAN standard of IEEE 802.15.6 but to a different IEEE standard, namely, the IEEE 802.15.4 standard [14].

Similarly, Subono et al. [15] proposed a time-scheduling approach for WBANs aimed at reducing energy consumption. A transceiver, with a data rate of 250 kbps and a transmit power of +18 dBm, was used to conduct the experiments. The transceiver used in that study was based on the IEEE 802.15.4 standard. In addition, the transmission power considered was excessively high for WBAN applications.

Some studies utilized FPGAs for hardware implementations. One such study was done by Priya et al. [16]. The study presented an FPGA-based hardware implementation of a narrowband (NB) PHY baseband transceiver for WBANs. They developed a transceiver using hardware description language and validated it using an ML605 evaluation board (Xilinx, Inc., San Jose, CA, USA). Their results indicated successful transmission and reception of 32 bytes at a frequency of 2.4 GHz and a speed of 121.4 kbps. However, no additional experiments were conducted to further validate the implementation, and there was also a lack of comparison with other studies.

In contrast to hardware implementations, some studies have focused solely on building WBAN simulations. We conducted one such study [7], in which we implemented the NB PHY for IEEE 802.15.6 in the ns-3 simulator. The study focused on capturing the key physical-layer features of WBANs, including the differential binary phase-shift keying (DBPSK) modulation scheme and Bose-Chaudhuri-Hocquenghem (BCH) codes. The study did not consider any real hardware implementations or include a comparison with hardware. The absence of experimental studies that directly compare WBAN simulations with practical hardware setups creates a discrepancy between theoretical performance analysis and the feasibility of real-world deployment.

Table 1 highlights the differences between existing studies and our evaluation of the WBAN PHY.

## 3. WBAN Simulation and Hardware Configuration

In this study, we compared hardware measurements with the corresponding WBAN implementation in ns-3 to assess the validity of simulation-based modeling. The evaluation focused on GMSK-based narrowband WBAN PHY behavior in realistic on-body and near-body scenarios. Although the hardware platform was not specifically designed for IEEE 802.15.6, it supports configurable PHY parameters that align with key parameters of the WBAN narrowband specification. This study considers WBAN operation in the 420–450 MHz NB band using Gaussian minimum shift keying (GMSK).

### 3.1. IEEE 802.15.6: WBAN Standard

**Figure 1:**
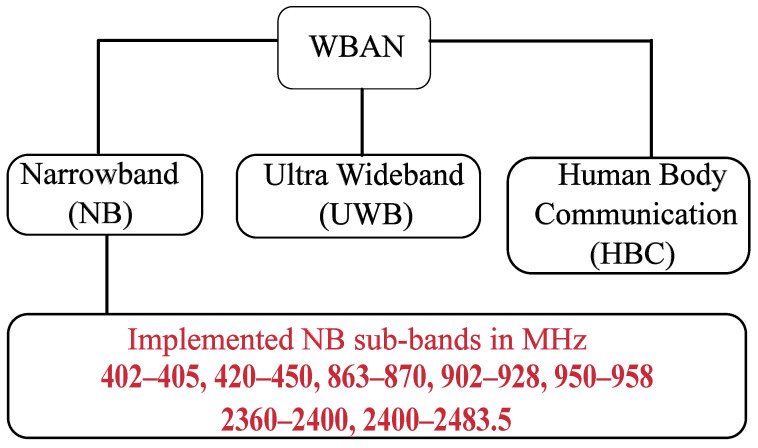
WBAN frequency bands [1].

The WBAN standard, also known as IEEE 802.15.6, operates on three main frequency bands: NB, ultrawideband (UWB), and human body communication (HBC), as illustrated in Figure 1. NB supports seven primary sub-frequencies, each with different data rates and modulation schemes. It also utilizes BCH codes for error correction.

UWB is designed to deliver robust performance for body area networks, offering high reliability and low complexity in ultra-low-power operation. Further, the HBC employs electric field communication (EFC) technology, allowing devices to transmit data through the human body without the need for wired or wireless connections.

### 3.2. WBAN Simulation Model

In this study, we used a discrete-event network simulator called ns-3 to simulate WBAN scenarios. Unlike other commonly used simulators, such as MATLAB, which focus on mathematical performance analysis, ns-3 models communication systems at the packet and protocol levels. In this approach, the simulation relies on scheduled network events, including packet transmissions, receptions, and routing decisions.

At the PHY within a discrete-event network simulator, communication is represented in an event-driven manner rather than through numerical computations. When a sender node transmits a packet, the simulator schedules its transmission and reception events for the packet. It then calculates the signal conditions at the receiving nodes by applying models for propagation, path loss, noise, and interference. This estimation provides metrics such as the received power, signal-to-noise ratio (SNR), and signal-to-interference-plus-noise ratio (SINR).

These computed values are then fed into an error model that probabilistically determines whether a packet has been successfully decoded or lost. This outcome then triggers subsequent events, such as the packet’s delivery to the next layer or its drop because of errors.

In this study, we used a WBAN simulation model developed in ns-3. The simulation included the WBAN PHY and a body propagation loss model. The WBAN PHY was built by considering the PHY parameters defined by the IEEE 802.15.6 standard. The primary goal of the body propagation loss model is to account for the propagation loss that occurs when a signal travels through various parts of the human body. The model calculates the path loss caused by the presence of the body in a network scenario.

We focused on the NB PHY of the WBAN. In our simulation, we implemented all seven NB frequency bands supported by the modulation schemes DBPSK and GMSK, along with their various data rates, in an additive white Gaussian noise (AWGN) environment. The study primarily focused on the 420–450 MHz frequency band for WBAN, which utilizes GMSK modulation. In Japan, the 420–450 MHz band is available for experimental purposes, making it suitable for WBAN deployment. In other countries, this frequency band is allocated to services such as military radars and government systems. Other modulation schemes defined by the WBAN standard, but not supported in our simulation, include DQPSK and D8PSK.

The PHY parameters considered for GMSK modulation by our simulation are listed in Table 2. The 420–450 MHz frequency band supports 12 channels. According to the WBAN standard, the bandwidth-time product (BT) for GMSK modulation was set to 0.5. The BT is a dimensionless parameter that governs the balance between bandwidth efficiency and inter-symbol interference. In WBANs, the preamble and physical layer convergence protocol (PLCP) headers are transmitted at different data rates than the protocol data unit (PSDU) to enable faster transmission of the preamble and PLCP. The implemented channel bandwidth is 320 kHz.

In our simulation, the probability of error for GMSK is given as [18]:

(1) $$P_{e}\approx \frac{1}{2}\mathrm{erfc}\left(\sqrt{\frac{\alpha E_{b}}{2N_{0}}}\right).$$

where erfc(·) is the error function, *a* = 0.78 for GMSK with BT= 0.5 [18].

Our simulation also supports BCH error-correcting codes for WBANs. Error correction is the process of detecting and correcting errors in transmitted messages to achieve reliable data transmission. For WBAN, two code rates were used: BCH (63, 51, t=2) for the PSDU and BCH (31, 19, t=2) for PLCP and preamble. These block codes are defined by their generator polynomial as (n,k) codes. For BCH (63, 51, t=2), n=63, which is the codeword length, and k=51, which is the length of the original information bits. In this case, t=2 indicates the capability to correct up to two bits of errors.

In our simulation, considering (n,k), the coded bit error probability is

(2) $$P_{e}\approx \frac{1}{2}\mathrm{erfc}\left(\sqrt{\frac{\alpha E_{b}}{2N_{0}}*\frac{k}{n}}\right).$$

The packet error rate for GMSK is simulated as

(3) $$PER=1-{\left(1-P_{e}\right)}^{b}.$$

where $P_{e}$ denotes the bit error probability of either the uncoded channel, as given in Equation (1) or the coded channel, as given in Equation (2), and *b* is the number of bits in the packet.

The 420–450 MHz WBAN band is unique in that the center frequency and channel spacing differ across its 12 channels. The center frequency defined by the WBAN standard is calculated as follows [1]

(4) $$f_{c}=420.30+0.50\times g_{1}\left(n_{c}\right)\;\left(\mathrm{MHz}\right),\;n_{c}=0,\ldots ,11$$

where $f_{c}$ denotes the center frequency, $n_{c}$ is the channel number, and $g_{1}\left(n_{c}\right)$ are the mapping functions and are defined as

(5) $$g_{1}\left(n_{c}\right)=\left\{\begin{array}{cc} n_{c} & 0\leq n_{c}\leq 1\\ n_{c}+6.875 & 2\leq n_{c}\leq 4\\ n_{c}+13.4 & n_{c}=5\\ n_{c}+35.025 & 6\leq n_{c}\leq 7\\ n_{c}+40.925 & 8\leq n_{c}\leq 9\\ n_{c}+47.25 & 10\leq n_{c}\leq 11. \end{array}\right.$$

Based on Equation (4) and the mapping functions defined in Equation (5), Table 3 provides a list of channel numbers along with their corresponding center frequencies and channel spacings utilized in this study. Each channel has a different spacing, which is unusual for conventional wireless systems that typically use uniform channel spacing. Although a nominal step size of 0.5 MHz is defined in Equation (4), the application of the mapping function $g_{1}\left(n_{c}\right)$ leads to a non-uniform distribution of channel frequencies. The effective separations between groups range from 0.5 MHz to 11.3125 MHz, ensuring spectral isolation and reducing interference. Notably, the last channel (Channel 11) has no subsequent channel, so no spacing is defined for it.

**Figure 2:**
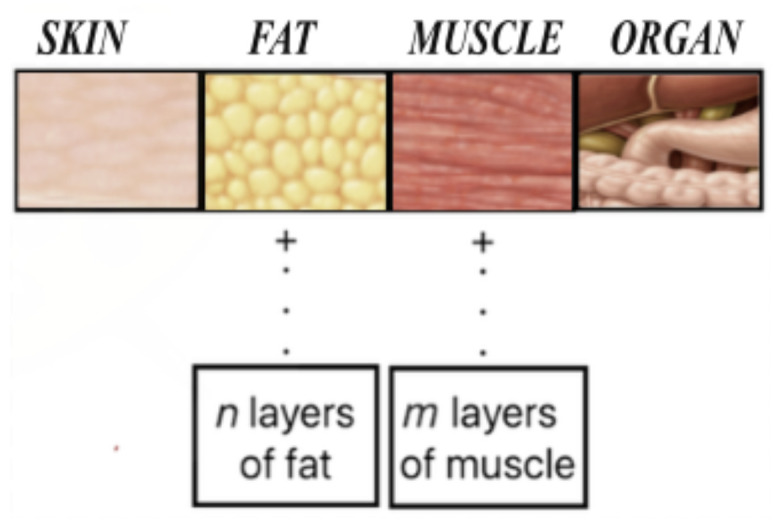
Layered structure for our body propagation loss model.

The body propagation loss model implemented in this study is part of our WBAN simulation, developed in ns-3. The body propagation loss model accounts for path loss caused by on-body (device placement on skin) and in-body (device placement in fat, muscle, or organ layers) communication. In this study, the model is used specifically to represent the additional attenuation associated with on-body device placement on the skin. Our model for human body propagation considers a layered structure. As shown in Figure 2, the outermost layer represents the skin, followed by layers of fat and muscle, and then internal organs. It is important to note that the thickness of the fat and muscle layers varies among individuals. To enhance the robustness of our propagation model, we offer the option to adjust the number of fat and muscle layers. It is important to note that the current body propagation loss model in this study remains simplified. It does not account for variations caused by factors such as age, gender, posture, tissue heterogeneity, or different skin-layer compositions.

Our body propagation loss model also considers the dielectric properties of human tissues. These properties significantly influence the propagation loss because they determine the electromagnetic absorption and flow of electrons within the body. Human tissue has two primary dielectric properties, namely, permittivity and conductivity. Permittivity quantifies a tissue’s ability to store an electric charge and resist an electric field, whereas conductivity quantifies how easily electrons can flow through the tissue. The path loss using the attenuation constant is calculated as [19]

(6) $$\alpha \approx \frac{520.8\pi \theta }{\sqrt{\epsilon_{r}}}*d.$$

where θ denotes the conductivity of the tissue, $\epsilon_{r}$ is the relative permittivity of the tissue, and *d* is the tissue thickness along the path of the signal.

Our WBAN simulation, consisting of the PHY and body propagation loss model, is publicly available and can be downloaded and implemented from the ns-3 app store website [17]. A detailed description of the download and implementation process is provided in [19].

The log-distance propagation loss model is an existing propagation loss model in ns-3. It is one of the most widely used models for simulating realistic wireless signal attenuation over distance. This model accounts for the decreases in signal strength because of the distance traveled by the signal. In this study, the log-distance propagation loss model is used to represent off-body propagation over air in the ns-3 scenarios. Furthermore, because ns-3 supports the stacking of multiple propagation loss models, this model is used together with the body propagation loss model to represent on-body communication conditions.

The log-distance propagation model used in ns-3 is expressed as [20],

(7) $$L=L_{0}+10n\log_{10}\left(\frac{d}{d_{0}}\right).$$

where *L* is the path loss (dB), $L_{0}$ is the path loss at reference distance (dB), *n* is the path loss distance exponent, $d_{0}$ is the reference distance (m), and *d* is the distance (m).

### 3.3. WBAN Hardware Platform and Configuration Settings

The hardware used in this study is the EFR32FG12 (Silicon Laboratories, Inc., Austin, TX, USA) [21], an MCU that interfaces with the BRD4258A mainboard (Silicon Laboratories, Inc., Austin, TX, USA). The EFR32FG12 supports operation in both the 2.4 GHz and sub-GHz bands. The MCU provides extensive PHY configurability and includes native support for various protocols based on IEEE 802.15.4, such as ZigBee and Thread. This configurability enables adaptation to different modulation and channel settings. It supports multiple modulation schemes, including 2-FSK, 4-FSK (with fully configurable shaping), OQPSK, GMSK, OOK, and ASK. The supported transmit power ranges from −26 to +20 dBm, and the supported packet size ranges from 7 to 64 bytes. The MCU features an integrated inverted-F PCB antenna, along with the option to connect an external Laird EXC470SM dipole antenna via the SMA antenna connector [21]. The Laird antenna has vertical polarization and a nominal impedance of 50 Ω, providing an effective gain of approximately 0 decibel-isotropic (dBi) [22].

The hardware configuration in this study was restricted to GMSK-based narrowband PHY operation. Although additional WBAN modulation schemes, such as DBPSK, were implemented in the ns-3 framework, they were not validated in hardware because the available platform does not provide native DBPSK support. Accordingly, the contribution of this paper should be interpreted as a practical validation of GMSK-based narrowband WBAN PHY behavior. This design choice preserves the validity of the simulation-to-hardware comparison under matched PHY settings while maintaining compatibility with commercially available hardware.

The WBAN hardware configurations were performed using the radio abstraction interface layer (RAIL), a customizable radio interface provided by Silicon Labs. RAIL allows users to customize their own radio interface by configuring PHY parameters. The RAIL interface is accessed through the Simplicity Studio application.

**Figure 3:**
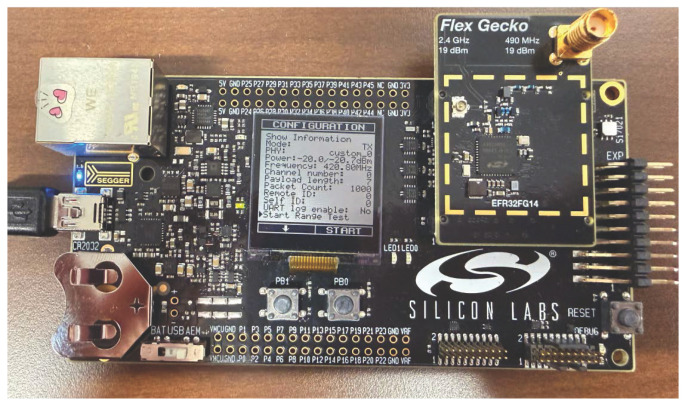
Hardware used for experimentation.

Figure 3 shows that EFR32FG12 is an MCU connected to the BRD4258A mainboard. The PHY parameters configured on the EFR32FG12 are identical to the PHY parameters set in the simulation. The frequency configuration was 420–450 MHz for GMSK modulation with a BT of 0.5. A bandwidth of 320 kHz was configured for a data rate of 151.8 kbps. Figure 3 also shows the WBAN PHY parameters configured on the hardware LCD screen.

## 4. Evaluation

This section presents the experimental setup and results from a series of off-body and on-body measurements conducted using an EFR32FG12 device. The objective was to assess the performance of the implemented WBAN PHY model in ns-3 against hardware measurements under realistic conditions. The evaluation also compares theoretical calculations, simulation-based predictions, and hardware-level behavior for WBANs.

We analyzed key performance metrics, including packet success rate, sensitivity, signal range across varying on- and off-body node placements, transmission distances, and power levels. By systematically comparing the measured results with the simulated outcomes, this evaluation highlights the reliability of the WBAN simulation model in ns-3. It also provides insight into the practical feasibility and limitations of the proposed platform.

### 4.1. Theoretical Calculations Against ns-3 Simulation

The ns-3 simulation implemented WBAN for GMSK modulation using the theoretical bit error probability. Because WBAN supports BCH error correction, the effect of BCH coding on bit error probability must also be considered. Error correction is the process of detecting and correcting errors in transmitted messages, aiming for reduced errors or even error-free data transmission. For NB transmissions, WBAN employs two BCH error-correcting code rates: BCH (63, 51, t=2) for the PSDU portion of the packet and BCH (31, 19, t=2) for the PLCP and preamble transmissions.

**Figure 4:**
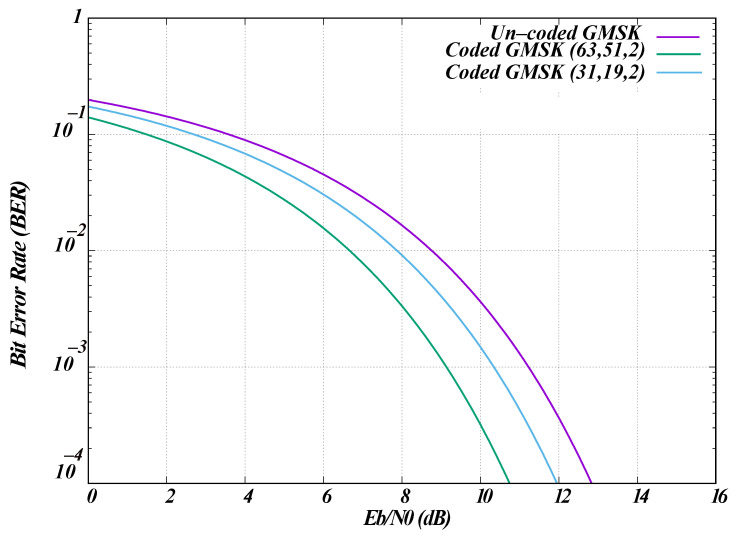
BCH coded and uncoded bit error probability for GMSK modulation.

Figure 4 shows the results of the calculation for the uncoded BER using Equation (1) and coded BER using Equation (2). The figure illustrates the relationship between BER and $E_{b}/N_{0}$ in decibels (dB) for uncoded GMSK, BCH (63, 51, t=2), and BCH (31, 19, t=2) code rates for GMSK modulation. Although the standard uses coded GMSK exclusively, the uncoded results are presented for comparison. In Figure 4, BCH (63, 51, t=2) demonstrates the lowest bit error probability compared with BCH (31, 19, t=2) and uncoded GMSK. Uncoded GMSK exhibits the highest error probability among the three because it lacks error correction capability. The simulation reproduces the exact results for uncoded GMSK reported in [18].

**Figure 5:**
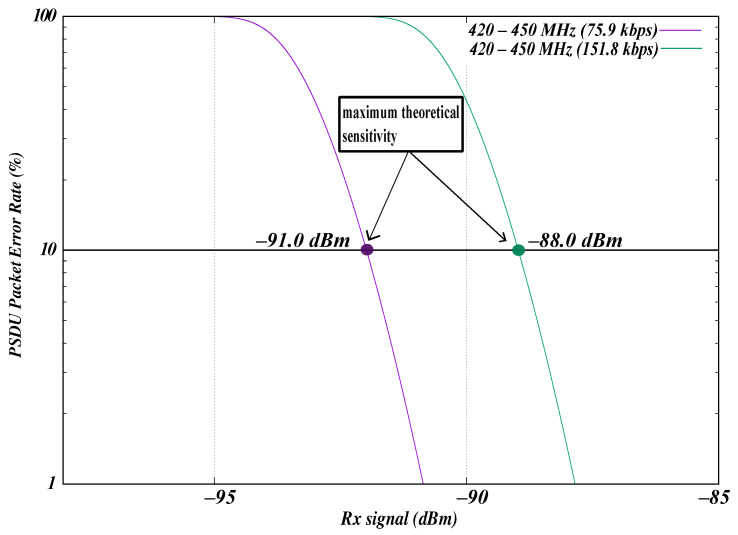
Packet error rate vs. Rx sensitivity for the 420–450 MHz WBAN band (standard specification).

To ensure alignment with the WBAN standard specifications, packet error rate (PER) and receiver (Rx) sensitivity were evaluated for the 420–450 MHz band. For WBAN PSDU data rates, the IEEE WBAN standard defines Rx sensitivity as the point at which the PER is less than or equal to 10%, using a PSDU of 255 octets in AWGN. Figure 5 illustrates the relationship between PER and Rx sensitivity for PSDU data rates in the 420–450 MHz band. The Rx sensitivity values are expressed in dBm. The PSDU PER shown in Figure 5 was calculated using Equation (3), based on the bit error probability under the WBAN standard condition of a 255-octet PSDU in an AWGN environment.

The Rx sensitivity was evaluated for all PSDU data rates using GMSK modulation, as shown in Table 2. The simulation results indicate that the Rx sensitivity for the frequency of the 420–450 MHz band and a PSDU data rate of 75.9 kbps is −91.0 dBm, whereas it is −88.0 dBm at a PSDU data rate of 151.8 kbps. The highest Rx sensitivity for this modulation and frequency band was observed at the lower data rate of 75.9 kbps.

The value of Rx sensitivity is influenced by factors such as data rates, modulation, environmental noise, antenna characteristics, and intersystem interference. This experiment was conducted with a fixed noise figure of 13 dB and an implementation loss of 6 dB, as recommended by the WBAN standard. The standard does not specify the rationale for these values. Under real-world conditions, the Rx sensitivity is expected to be significantly higher than the values observed in this experiment. This is because manufacturers strive to create highly sensitive receivers to ensure that their devices can operate effectively across a variety of scenarios. Greater sensitivity allows for the reception of weaker signals, thus extending the communication range.

Although simulation environments allow users to adjust sensitivity parameters based on their specific requirements, the sensitivity of real-world hardware is fixed and cannot be modified by users. As a result, manufacturers focus on optimizing receiver sensitivity, leading to real-world devices that demonstrate enhanced sensitivity compared to their simulated or theoretical counterparts.

**Figure 6:**
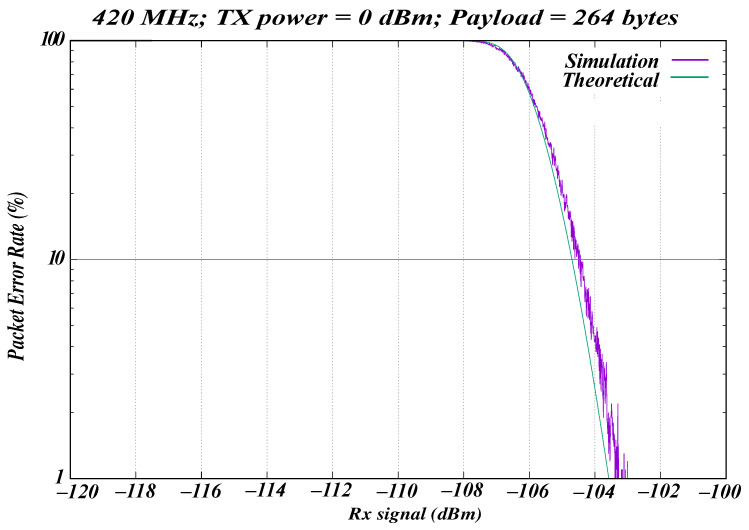
Theoretical vs. simulation for the 420–450 MHz WBAN band.

To further justify the reliability of the simulation model, theoretical calculations were compared with the ns-3 simulation results. Figure 6 shows the relationship between PER and Rx sensitivity for the 420–450 MHz WBAN band under simulation and theoretical scenarios. The theoretical PER is obtained analytically from Equation (3) and therefore follows a smooth deterministic trend. By contrast, the simulated PER is derived from finite packet transmissions in ns-3, where packet reception is determined probabilistically according to a random component that simulates the variability of channel conditions. This leads to small fluctuations in the simulated PER around the theoretical curve. The same PHY parameters were used for both theoretical and simulation scenarios.

The PSDU payload size considered in this experiment was 264 bytes, consisting of seven bytes of the MAC header, 255 bytes of the MAC frame, and two bytes of the frame check sequence (FCS). The transmission (Tx) power was 0 dBm. The Rx sensitivity considered was −118.92 dBm, representing the maximum theoretical possible sensitivity at 420–450 MHz.

The maximum theoretically possible sensitivity at 420–450 MHz with a bandwidth of 320 kHz was calculated using

(8) $$P_{n}=10\log_{10}\left(\frac{k\cdot B\cdot T}{1\;\mathrm{mW}}\right).$$

where *k* is the Boltzmann constant, $1.38064852\times 10^{-23}\;{\mathrm{JK}}^{-1}$, *T* is the temperature (set to 290 K), and *B* is the bandwidth of the frequency at which the Rx sensitivity is being measured, specified as 320 kHz in Table 2.

The theoretical scenario provides a reference PER for the ns-3 simulation at a given Rx signal strength. Because no interference was present in the theoretical setup, PER was deterministic for a fixed SNR ratio. The ns-3 simulation scenario consisted of two nodes using the log-distance propagation loss model. These nodes operated in the 420–450 MHz band on channel 0 and transmitted 1000 packets. As shown in Figure 6, the experimental and theoretical results are closely aligned, demonstrating the consistency of the simulation model.

### 4.2. Simulation vs. Hardware Evaluation for Off-Body WBAN

#### 4.2.1. Experimental Setup

**Figure 7:**
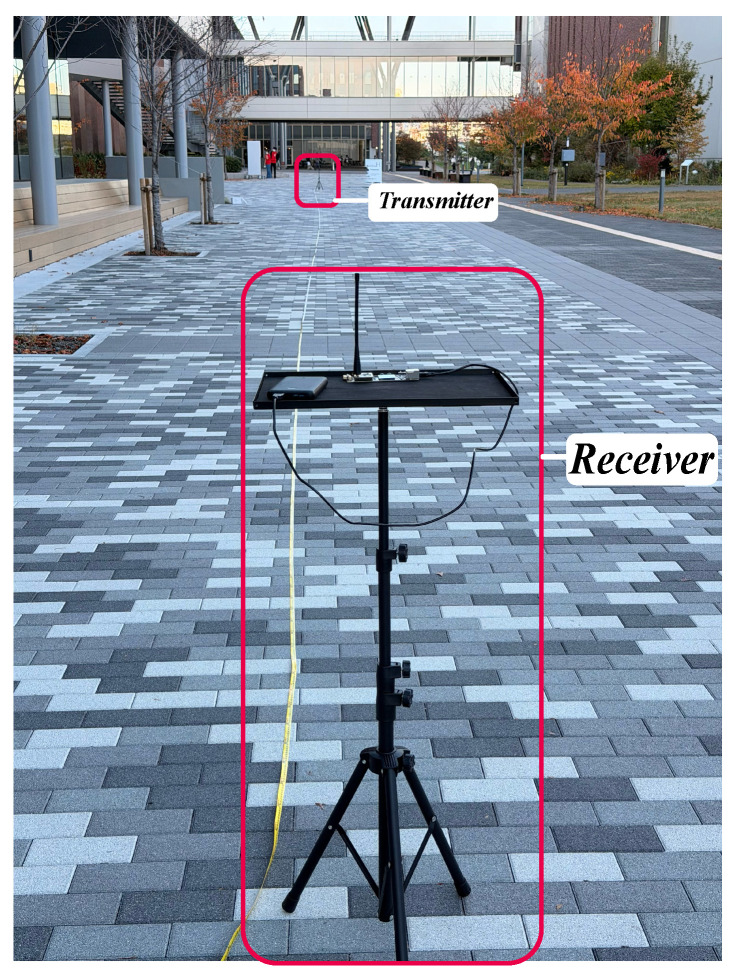
Off-body experimental set-up.

The EFR32FG12 platform with an external Laird EXC470SM dipole antenna (Laird Technologies, Inc., Akron, OH, USA) with an effective gain of approximately 0 dBi was used for the off-body experiments. The off-body experiment was performed outdoors, and the experimental setup is shown in Figure 7. Two devices were placed at variable distances of up to 100 m with no human body present in the signal transmission path.

#### 4.2.2. Evaluation

Although WBAN applications typically target short-range communication, extended-distance measurements were included here to observe packet success rate (PSR) degradation trends under the lowest transmit powers supported by the hardware platform.

**Figure 8:**
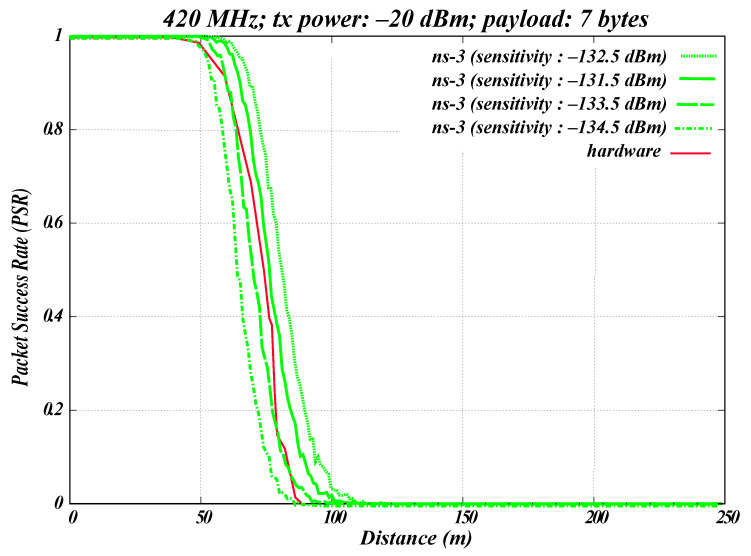
Simulation vs. hardware for off-body WBAN.

To test our simulation model, we compared the results for the 420–450 MHz WBAN band with GMSK modulation in ns-3 with real hardware measurements, as shown in Figure 8. The figure shows the relationship between the PSR and the distance (m) that a transmitted signal travels. The PHY parameters were identical for both the ns-3 simulation and the hardware setup. The parameter values used in this experiment are listed in Table 4.

Hardware experiments were conducted using the EFR32FG12 radio by Silicon Labs, as explained in Section 3. The radio operated at 420.80 MHz, corresponding to channel 0 of the frequency band. The hardware was configured with a Tx power of −20 dBm, reflecting the typical low-power operation of WBAN devices (0 to −40 dBm) [23]. A payload size of seven bytes was used, and 1000 packets were transmitted. Measurements were recorded at 10 m increments up to 100 m, and Figure 8 shows the average of five radio runs. These PHY parameters are also displayed on the radio screen, as shown in Figure 3.

In the ns-3 simulation, two nodes were configured with the same parameters as the hardware: a frequency of 420.80 MHz and a Tx power of −20 dBm, transmitting 1000 packets of seven bytes each. Notably, the receiver sensitivity of the radio for GMSK modulation is not specified in the data sheet. Therefore, a small sensitivity sweep analysis was conducted in ns-3 using effective system-level sensitivity values from −134.5 dBm to −131.5 dBm under matched PHY settings.

The sensitivity used in ns-3 should not be interpreted as the intrinsic receiver sensitivity of the EFR32FG12 chipset; rather, it captures antenna-related and implementation-specific effects that are not explicitly modeled in ns-3. Although this value is lower (i.e., indicates higher sensitivity) than typical standard expectations, it was introduced in the simulation because the hardware setup included an external antenna and other practical effects beyond the simplified simulation model. For each sensitivity setting, the ns-3 results shown in Figure 8 represent the average of five simulation runs.

In addition, a log-distance propagation loss model was used in the simulation to reflect signal propagation through air in the hardware scenario. As shown in Figure 8, an ns-3 sensitivity of −133.5 dBm provides the closest overall agreement with the hardware trend across the measured distance range. The sensitivity value of −134.5 dBm is overly optimistic, resulting in consistently higher PSR than that observed in the hardware measurements. This indicates that the simulated receiver is unrealistically capable of decoding weaker signals than the actual device.

On the other hand, sensitivities of −132.5 dBm and −131.5 dBm underestimate the receiver performance. These values lead to lower PSR compared to the hardware results, suggesting that the simulated receiver becomes insensitive too early and fails to capture packets that are successfully received in the real system.

Based on this sensitivity sweep, the following WBAN experiments use an effective system-level sensitivity of −133.5 dBm in ns-3, as it provides the closest approximation to the observed hardware behavior under the evaluated off-body conditions.

**Figure 9:**
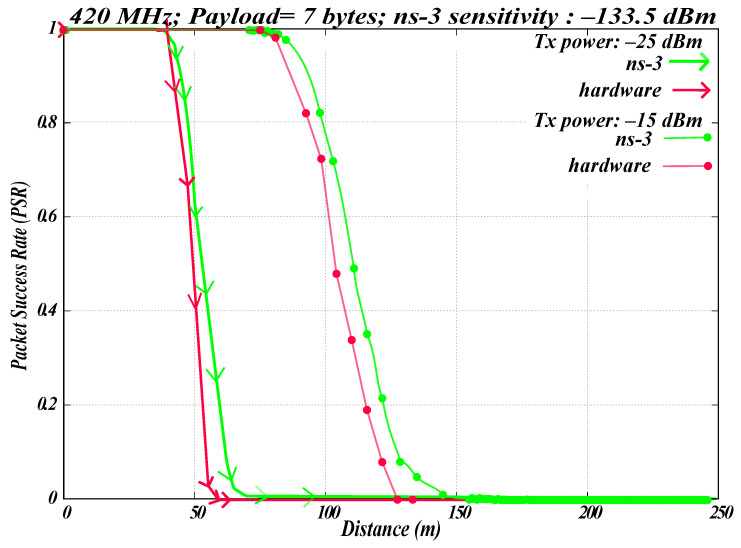
Simulation vs. hardware for off-body WBAN at different Tx powers.

Our WBAN simulation model in ns-3 is configurable, allowing adjustment of PHY parameters such as data rates and Tx power. Figure 9 presents the results of the off-body WBAN comparison at different Tx powers at 420.80 MHz. The experimental setup is shown in Figure 7. Hardware experiments were conducted using the EFR32FG12 module.

A payload size of seven bytes was used, and 1000 packets were transmitted. Measurements were recorded at 10 m intervals up to 150 m. Figure 9 represents the average of five hardware runs. In the ns-3 simulation, the same parameters were used: a frequency of 420.80 MHz, 1000 transmitted, and a payload of seven bytes with a sensitivity consideration of −133.5 dBm. The ns-3 results displayed in Figure 9 show the average of five simulation runs.

As shown in Figure 9, for the hardware setup, the signal reaches a distance of 55 m with a transmission power of −25 dBm, whereas at −15 dBm, the signal extends up to 125 m. Notably, in ns-3, the signal range extends approximately 10 m beyond the hardware results. Figure 9 also shows minimal differences in PSR between ns-3 and hardware results, indicating a close agreement. Together with the results in Figure 9 alongside the earlier findings in Figure 8, this agreement supports the validity of the ns-3 WBAN simulation model under the assumed sensitivity conditions.

### 4.3. Cross-Standard Validation Using LR-WPAN (IEEE 802.15.4)

#### 4.3.1. Experimental Setup

**Figure 10:**
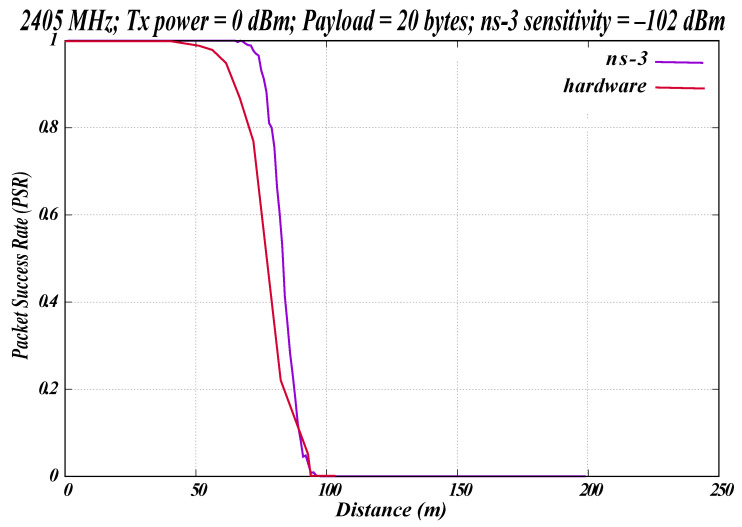
Simulation vs. hardware for 2.4 GHz LR-WPAN.

EFR32FG12 hardware with an integrated inverted-F PCB antenna was used for off-body cross-standard validation experiments, as shown in Figure 10. The off-body experiment was performed outdoors, and the experimental setup is shown in Figure 7. Two devices were placed at variable distances up to 100 m. No human body was present in the signal transmission path during the off-body experiments.

#### 4.3.2. Evaluation

To complement the WBAN evaluation, the same hardware platform was also analyzed under LR-WPAN (IEEE 802.15.4) and its corresponding ns-3 model. The LR-WPAN experiment is included here as a cross-standard control experiment using the same hardware platform. Its purpose is not to shift the focus away from WBAN but to provide an additional simulation-to-hardware reference point based on a mature ns-3 standard implementation. The EFR32FG12, depicted in Figure 3, was configured for LR-WPAN as defined by IEEE 802.15.4 [14]. Because the hardware provides native, standard-compliant support for LR-WPAN, no custom PHY reconfiguration was needed. By contrast, the WBAN experiments required a custom configuration of parameters such as frequency band, bandwidth, center frequency, channel number, data rate, header, and frame size. Observing similar trends across both WBAN and LR-WPAN evaluations strengthens confidence in the validity of the WBAN simulation-to-hardware comparison.

Figure 10 shows the relationship between the PSR and the distance the signal travels in meters. The LR-WPAN simulation experiments were conducted using the existing LR-WPAN module in ns-3. The PHY parameters remained consistent for both ns-3 and the hardware experiments. The parameter values used in this experiment are listed in Table 5.

The radio operates on channel 11 at the 2405 MHz frequency band. The modulation technique employed in this experiment was offset quadrature phase-shift keying (OQPSK), as specified in the LR-WPAN standard. The hardware operated at a Tx power of 0 dBm and used a payload length of 20 bytes, comprising seven bytes for the MAC header, 11 bytes for the MAC frame, and two bytes for the FCS. A total of 1000 packets were transmitted. Measurements were recorded at 10 m increments up to 100 m; Figure 10 displays the average of five hardware runs.

In the ns-3 simulation, two nodes were modeled using the same parameters as the hardware. We considered a sensitivity of −102 dBm in the simulation, as specified in the EFR32FG12 data sheet for OQPSK modulation [21]. A log-distance propagation loss model was incorporated to represent signal propagation through air, consistent with the hardware scenario. At 80 m, the hardware exhibited a PSR of approximately 30%, whereas ns-3 maintained a PSR of 80%. As discussed in the previous WBAN experiment, the higher PSR observed in ns-3 can be attributed to the idealized and simplified simulation conditions, whereas the hardware is impacted by real-world interference.

### 4.4. On-Body vs. Off-Body Performance Analysis Using Hardware and Body Propagation Loss Model in ns-3

#### 4.4.1. Experimental Setup

**Figure 11:**
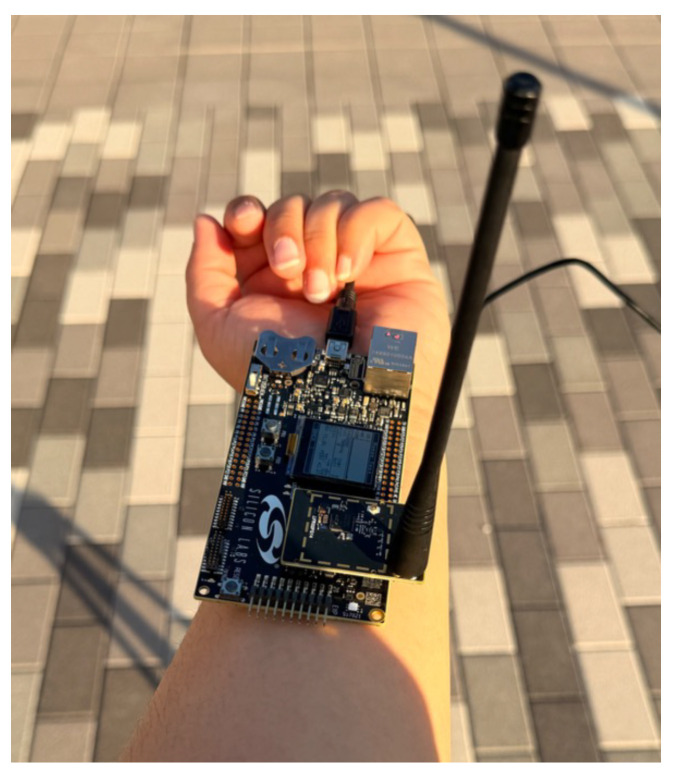
On-body WBAN experimental setup.

The EFR32FG12 platform with an external Laird EXC470SM dipole antenna with an effective gain of approximately 0 dBi was used for the on-body hardware experiments. The on-body experiment was performed outdoors. The experimental setup is shown in Figure 11. The figure shows one device placed on the body, specifically on the wrist, and another positioned around the human body [11]. The second device was positioned at variable distances up to 110 m. One subject participated in the on-body experiment.

#### 4.4.2. Evaluation

**Figure 12:**
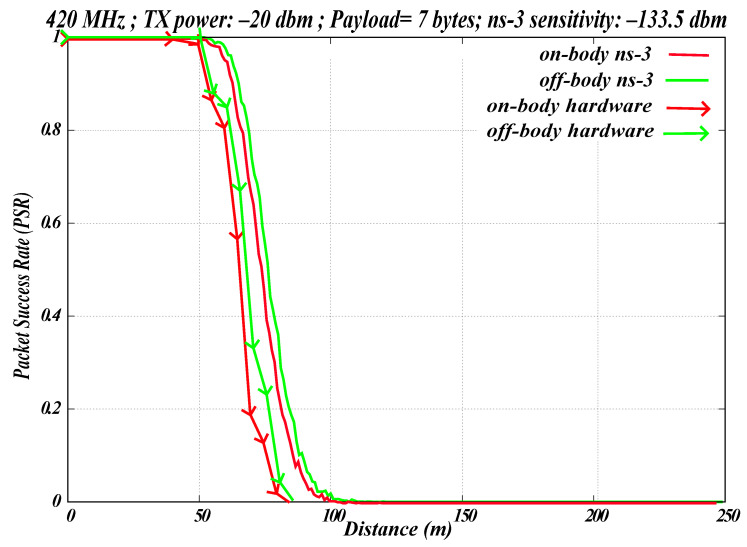
Simulation vs. hardware for on-body WBAN.

The ns-3 model for WBAN includes a body propagation loss model that was developed as a part of our previous study. The body propagation loss model accounts for the effects of human body presence on the signal transmission. It accounts for path loss in scenarios where one node is placed on the skin and the other is near the body. As shown in Figure 12, the results of the body propagation loss model in ns-3 were compared with hardware measurements for GMSK modulation.

The PHY parameters set for the hardware in this experiment matched those used in ns-3, as shown in Table 4. In this experiment, the EFR32FG12 radio operated at a frequency of 420.80 MHz, corresponding to channel 0 of the frequency band. The hardware was configured with a Tx power of −20 dBm, reflecting the typical low-power operational range of WBAN devices, and transmitted 1000 packets with a payload size of seven bytes. These PHY parameters are also displayed on the radio screen, as shown in Figure 3. In the ns-3 simulation, the same PHY parameters were used: 420.80 MHz, −20 dBm Tx power, 1000 packets, and a payload size of seven bytes. A sensitivity of −133.5 dBm was considered.

Four scenarios were considered in this experiment. The first scenario was off-body hardware, involving the EFR32FG12 platform, where the transmitter and receiver were positioned at varying distances to measure PSR. This scenario did not include a human body in the signal path, as shown in Figure 7. The second scenario was on-body hardware, also involving the EFR32FG12, where the transmitter was placed around the human body and the receiver on the wrist, as shown in Figure 11. Measurements for these two scenarios were recorded at 10 m increments up to 110 m, and Figure 12 shows the average of five hardware runs.

The third scenario was off-body ns-3, which used the ns-3 WBAN simulation model, where a transmitter and receiver were placed at varying distances, and PSR was calculated based on propagation over air using the log-distance propagation loss model in ns-3. This scenario did not include a human body in the signal path. The final scenario was on-body ns-3, which also employed the ns-3 WBAN simulation model, in which the transmitter was placed around the human body and the receiver on the skin. In this case, the PSR was calculated with the body propagation loss model, which accounts for propagation loss owing to the human body, together with the log-distance propagation loss model for propagation over air. The ns-3 results displayed in Figure 12 show the average of five simulation runs.

The values of permittivity and conductivity used in this study are listed in Table 6. The attenuation contributed by the dry-skin layer alone, under the assumed dielectric parameters and thickness, is approximately 0.2176 dB [24,25]. This value should not be interpreted as the total excess loss of a practical on-body propagation channel, which may be substantially larger in practice owing to posture, body shadowing, antenna placement, tissue heterogeneity, and environmental effects.

When comparing the PSR difference at 40% between ns-3 (with and without the body) and hardware (with and without the body), similar trends are observed, as illustrated in Figure 12. Notably, the hardware achieved a maximum distance of 80 m, whereas the simulation reached up to 100 m. This discrepancy is attributed to the hardware scenarios being susceptible to other environmental interferences that are not considered in the simulation, which allows the simulated results to extend further.

The communication distances observed in Figure 8, Figure 9 and Figure 12 extend beyond those of typical WBAN use cases. This does not imply that practical WBAN systems are intended to operate over such ranges. Rather, these measurements reflect the minimum transmit-power constraints of the available hardware platform, which supports power levels only down to −26 dBm. Lower-power experiments more representative of sub-3 m WBAN operation remain for future work.

Unless otherwise stated, the reported hardware and simulation results represent averages over five runs. The present evaluation is intended to examine the consistency of performance trends between simulation and hardware under matched PHY settings; a more extensive statistical characterization is left for future work.

## 5. Limitations and Future Work

- The study focuses exclusively on PHY-layer validation of NB GMSK modulation for both hardware and simulation. Other WBAN modulation schemes, such as DBPSK, were implemented in simulation but not evaluated in hardware due to the platform’s lack of native support.
- The contribution of the paper is a practical validation of GMSK-based narrowband WBAN PHY behavior, not a comprehensive validation of all IEEE 802.15.6 PHY modes.
- The reported value of 0.2176 dB represents the attenuation contribution of the modeled dry-skin layer under the assumed dielectric parameters and thickness, not the total excess loss of a practical on-body propagation channel.
- The current body propagation loss model is a preliminary framework in ns-3, not an empirical description of on-body propagation.
- The simulation does not include fading, body shadowing, multipath propagation, or other environmental interference effects, which may account for discrepancies between simulation results and hardware measurements.
- The use of an effective system-level sensitivity of −133.5 dBm in the ns-3 model should not be interpreted as the intrinsic receiver sensitivity of the chipset.
- A small off-body sensitivity sweep analysis was conducted in this study to support the selection of the effective system-level sensitivity used in ns-3. However, a more extensive sensitivity analysis across additional scenarios and parameters remains for future work.
- The study utilized a relatively large sensor board, which does not fully reflect the compact and body-conformal designs expected in practical WBAN deployments. Therefore, the current serves as a proof-of-concept for PHY-level validation, not as a final wearable hardware design.
- Future work will aim to address these limitations, extend validation to more WBAN layers and modulation modes, improve the body propagation model, and explore hardware platforms that better support IEEE 802.15.6-oriented WBAN experiments.

## 6. Conclusions

The primary focus of this study was to compare WBAN hardware results with those of a WBAN simulation. An MCU was configured to evaluate hardware performance in WBANs. An ns-3-based WBAN simulation implementing GMSK modulation compliant with the WBAN standard was developed. The simulation also included the body propagation loss model, which calculates path loss when the human body is present in the signal path.

The simulation analysis reveals that GMSK with BCH (63, 51, t = 2) coding achieves the lowest bit error probability compared to BCH (31, 19, t = 2) and uncoded GMSK, highlighting the significance of channel coding in improving communication reliability. It is also observed that the highest receiver sensitivity for GMSK in the 420–450 MHz band occurs at the lower data rate of 75.9 kbps. The results indicate that the simulation aligns with the theoretical calculations mentioned in the WBAN standard.

The experiments included both on- and off-body WBAN scenarios based on realistic conditions. For the on-body experiments, one device was placed on a subject’s wrist, a common wearable location, whereas the second device was positioned near the body. Identical PHY parameters were analyzed in both the simulation and hardware to ensure consistency. The findings also show close agreement between the simulation and hardware results, supporting the suitability of the proposed WBAN simulation model for practical applications.

## Figures and Tables

**Table 1:** Summary of literature review.

Literature	Implementation	Simulation vs. Hardware	WBAN Compliant	Source Code Availability
Kasun et al. [11]	Custom-built hardware	No	Yes	No
Su et al. [12]	Custom-built hardware	No	Yes	No
Isak et al. [13]	Empirical dataset and simulation	Yes	Yes	No
Woosik et al. [10]	Commercial hardware	No	No	No
Subono et al. [15]	Commercial hardware	No	No	No
Priya et al. [16]	Custom-built hardware	No	Yes	No
Our Implementation	Commercial hardware and simulation	Yes	Yes	Yes [17]

**Table 2:** PHY parameters considered for GMSK modulation.

Parameter	Value
Modulation type	GMSK (M = 2)
Frequency	420–450 MHz
Number of channels	12 (0 to 11)
BT	0.5
Preamble and PLCP data rate	57.5 kbps
PSDU data rates	75.9, 151.8, 187.5 kbps
Channel bandwidth	320 kHz

**Table 3:** GMSK center frequency calculations.

Channel Number	Center Frequency (MHz)	Channel Spacing (MHz)
0	420.30	0.50
1	420.80	3.9375
2	424.7375	0.50
3	425.2375	0.50
4	425.7375	3.7625
5	429.50	11.3125
6	440.8125	0.50
7	441.3125	3.45
8	444.7625	0.50
9	445.2625	3.6625
10	448.925	0.50
11	449.425	Not applicable

**Table 4:** WBAN parameters considered for ns-3 and hardware.

Parameter	Value (ns-3 and Hardware)
Frequency	420–450 MHz
Channel number	0
Channel bandwidth	320 kHz
Modulation	GMSK (BT = 0.5)
Data rate	151.8 kbps
Packet size	7 bytes
Transmission power	−20 dBm

**Table 5:** LR-WPAN parameters considered for ns-3 and hardware.

Parameter	Value (ns-3 and Hardware)
Frequency	2400 MHz
Channel number	11
Modulation	OQPSK
Data rate	250 kbps
Packet size	20 bytes
Transmission power	0 dBm
Sensitivity	−102 dBm [21]

**Table 6:** Dielectric parameters used in the body propagation loss model (on-body).

Parameter	Value
Tissue type	Dry Skin
Frequency	420–450 MHz
Conductivity (θ) [26]	0.69652
Permittivity ($\epsilon_{r}$) [26]	46.345
Layer thickness	0.0013 m
Propagation loss (α)	0.2176 dB

## Data Availability

The WBAN simulation is publicly available on the ns-3 app store [17].
